# Epigenetic analysis of high and low motile sperm populations reveals methylation variation in satellite regions within the pericentromeric position and in genes functionally related to sperm DNA organization and maintenance in *Bos taurus*

**DOI:** 10.1186/s12864-019-6317-6

**Published:** 2019-12-06

**Authors:** Emanuele Capra, Barbara Lazzari, Federica Turri, Paola Cremonesi, Antônia Moemia Rodrigues Portela, Paolo Ajmone-Marsan, Alessandra Stella, Flavia Pizzi

**Affiliations:** 1 Istituto di Biologia e Biotecnologia Agraria, Consiglio Nazionale delle Ricerche, via Einstein, 26900 Lodi, Italy; 2 Department of Animal Science, Federal University of Ceará, Fortaleza, Brazil; 3 Department of Animal Science, Food and Nutrition - DIANA, Università Cattolica del Sacro Cuore, Piacenza, Italy; 4 Proteomics and Nutrigenomics Research Center - PRONUTRIGEN, Università Cattolica del Sacro Cuore, Piacenza, Italy

**Keywords:** Sperm, Motility, Methylation, Epigenetic, Satellite

## Abstract

**Background:**

Sperm epigenetics is an emerging area of study supported by observations reporting that abnormal sperm DNA methylation patterns are associated with infertility. Here, we explore cytosine-guanine dinucleotides (CpGs) methylation in high (HM) and low motile (LM) *Bos taurus* sperm populations separated by Percoll gradient. HM and LM methylation patterns were investigated by bisulfite sequencing.

**Results:**

Comparison between HM and LM sperm populations revealed that methylation variation affects genes involved in chromatin organization. CpG Islands (CGIs), were highly remodelled. A high proportion of CGIs was found to be methylated at low/intermediate level (20–60%) and associated to the repetitive element BTSAT4 satellite. The low/intermediate level of methylation in BTSAT4 was stably maintained in pericentric regions of chromosomes. BTSAT4 was hypomethylated in HM sperm populations.

**Conclusions:**

The characterization of the epigenome in HM and LM *Bos taurus* sperm populations provides a first step towards the understanding of the effect of methylation on sperm fertility. Methylation variation observed in HM and LM populations in genes associated to DNA structure remodelling as well as in a repetitive element in pericentric regions suggests that maintenance of chromosome structure through epigenetic regulation is probably crucial for correct sperm functionality.

## Background

Male infertility is a complex disorder affecting humans as well as other animals. Infertility is partially explained by physiological and biochemical factors, such as low sperm counts and poor sperm quality. The genetic basis of male infertility accounts for about 15% of infertile cases [1, 2]. The etiology of this disorder remains unclear both in human and other species. For example, bulls considered of high-merit based on different sperm traits such as spermatozoa motility and morphology, are sometimes unable to produce successful full-term pregnancies [3, 4]. Different molecular parameters related to sperm nuclear and mitochondrial DNA, plasma membrane and lipid composition affect the ability of spermatozoa to fertilize oocytes and contribute to normal embryo development [5–7]. Therefore, much remains to be understood and novel molecular approaches may help to unravel the molecular basis of infertility.

Among the known epigenetic processes in mammalian cells, DNA methylation has been identified as an important regulatory mechanism of genome function in normal embryonic development, X-chromosome inactivation and genomic imprinting [8, 9]. DNA methylation of the 5-carbon position in cytosine residues was reported to be predominantly present in cytosine-guanine dinucleotides (CpG) and especially in GC rich regions called CpG islands (CGIs) [10]. CGIs methylation in different genomic features impacts gene expression, i.e., promoter demethylation is associated with gene expression, while methylation in gene bodies influences splicing [11]. Methylation is also observed in Repetitive Elements (RE) of adult cells playing a role in the maintenance of chromosome structure and genome integrity [12].

Sperm epigenetic marks are unique, thus the factors that determine the patterns of DNA methylation differ between male germ cells and somatic cells. Although RE are highly methylated in both germ and somatic cells, elements from several subfamilies show different levels of methylation in the two cell types [13]. Centromeric regions in spermatogonia are known to be less methylated compared to somatic tissues [14]. This methylation pattern is supposed to play a role in germ-cell chromatin organization, rather than in the control of gene expression [15]. Most of the epigenetic signatures in germ cells are erased after conception from the morula stage to the blastocyst stage in the inner cell mass (ICM); successively, a sharp increase in the level of methylation in the embryo is observed following implantation [16, 17]. However, a proper regulation of epigenetic processes during spermatogenesis is necessary to ensure embryonic development in addition to sperm function. The level of DNA methylation of round spermatid was reported to be different from that of mature spermatozoa. Round spermatid rather than mature spermatozoa microinsemination was also observed to profoundly influence epigenetic marks in the embryo, thus affecting embryonic development and male fertility [18].

Aberrant locus specific or global methylation has been associated to abnormal semen parameters, as well as to male infertility. Oligospermic patients were reported to present a hypomethylation or unmethylation pattern at the H19 gene encoding insulin-like growth factor 2 (IGF2) imprinting control region 1 (ICR1); furthermore, hypermethylation at the Mesoderm-specific transcript (MEST) imprinted locus as well as a reduced sperm quality, as compared with normozoospermic men [19]. Broad DNA hypermethylation across many loci, also including the Satellite 2 repetitive element, was associated to poor sperm concentration and motility and to morphology alterations in abnormal human sperm [20]. The level of DNA methylation in human sperm, determined through an ELISA-like method, was correlated to conventional sperm parameters, e.g. concentration and motility, as well as sperm chromatin and DNA integrity, but not to sperm viability and morphology [21]. DNA methylation in human spermatozoa was higher in low quality spermatozoa [22]. Pyrosequencing analysis of long interspersed elements (LINE) in human sperm, after bisulfite conversion, estimated an overall global methylation of about 75%, increasing with age. At the same time, targeted bisulfite sequencing of different selected genes showed a lower methylation level with a strong trend toward age-associated hypomethylation in some genomic regions [23]. Targeted bisulfite sequencing also revealed different levels of methylation in the promoter regions between high and low motile human sperm [24].

In farm animals, several studies showed altered sperm methylation to be associated with male infertility. DNA methylation pattern were found to be different between spermatozoa from high-fertile and sub-fertile buffalo bulls [25]. Recently, assessment of the epigenetic signature of bull spermatozoa using a human DNA methylation microarray [26] and Methyl-Binding Domain (MBD) Sequencing [27] revealed differentially methylated CpG sites and regions associated to bull fertility rate.

In the present study, the 5-methyl cytosine variations in CpGs were evaluated in high and low motility bull sperm populations following methyl enrichment and bisulfite sequencing approach. The objective is to produce a genome-wide methylation profile in the two populations, and to identify differential epigenetic signatures between high (HM) and low motile (LM) sperm.

## Results

### Isolation of spermatozoa and evaluation of sperm characteristics

Sperm cells were successfully fractionated into HM and LM populations; a significant (*P* < 0.05) improvement of several sperm quality parameters was observed in HM population in comparison to semen at thawing considering the following parameters: straight-line velocity VSL, curvilinear velocity VCL, average path velocity VAP and amplitude of lateral head displacement ALH variables [VSL (μm/s): 46.08 ± 4.11, 61.24 ± 2.91; VCL (μm/s): 76.35 ± 6.02, 110.37 ± 4.25; VAP (μm/s): 55.38 ± 4.27, 74.02 ± 3.01; ALH (μm) 2.53 ± 0.14, 3.72 ± 0.10; respectively in semen at thawing and HM population (Additional file 1).

### Sequencing statistic and CpG methylation distribution

Since methylation levels in sperm are expected to be generally high [13], the Methyl-binding domain (MDB) approach was used to select hypermethylated regions [28]. Bisulfite sequencing was then applied to the methylation-enriched genomic fraction to investigate CpG methylation level at single base resolution in the highly methylated regions. To exclude the presence of technical biases caused by unbalanced sequencing between groups of samples due to MBD enrichment, we evaluated cytosine coverage consistence between the HM and LM groups; no technical bias related to the combined approach (MDB and bisulfite sequencing) was observed (Additional file 2).

The average number of reads per sample was 28.1 M (range: 13.2 M–37.5 M). Mapping efficiency was high for all samples (83.1–90.6%). After calculating cytosine methylation conversion, a high percentage (93.7%) of the cytosines in the CpG enriched regions was methylated in both sperm populations (see Additional file 3 for statistics). After applying a threshold of at least 5X coverage per cytosine, a total of 26.6 M methylated regions (MR) (100 bp tiles with sliding window size of 100 bp) were identified spanning across the whole bovine genome. Among these, 1,086,748 methylated regions (MRs), shared between at least 3 out of 4 for both HM and LM sperm populations, were selected for DNA cytosine methylation profile comparison.

**Fig. 1:**
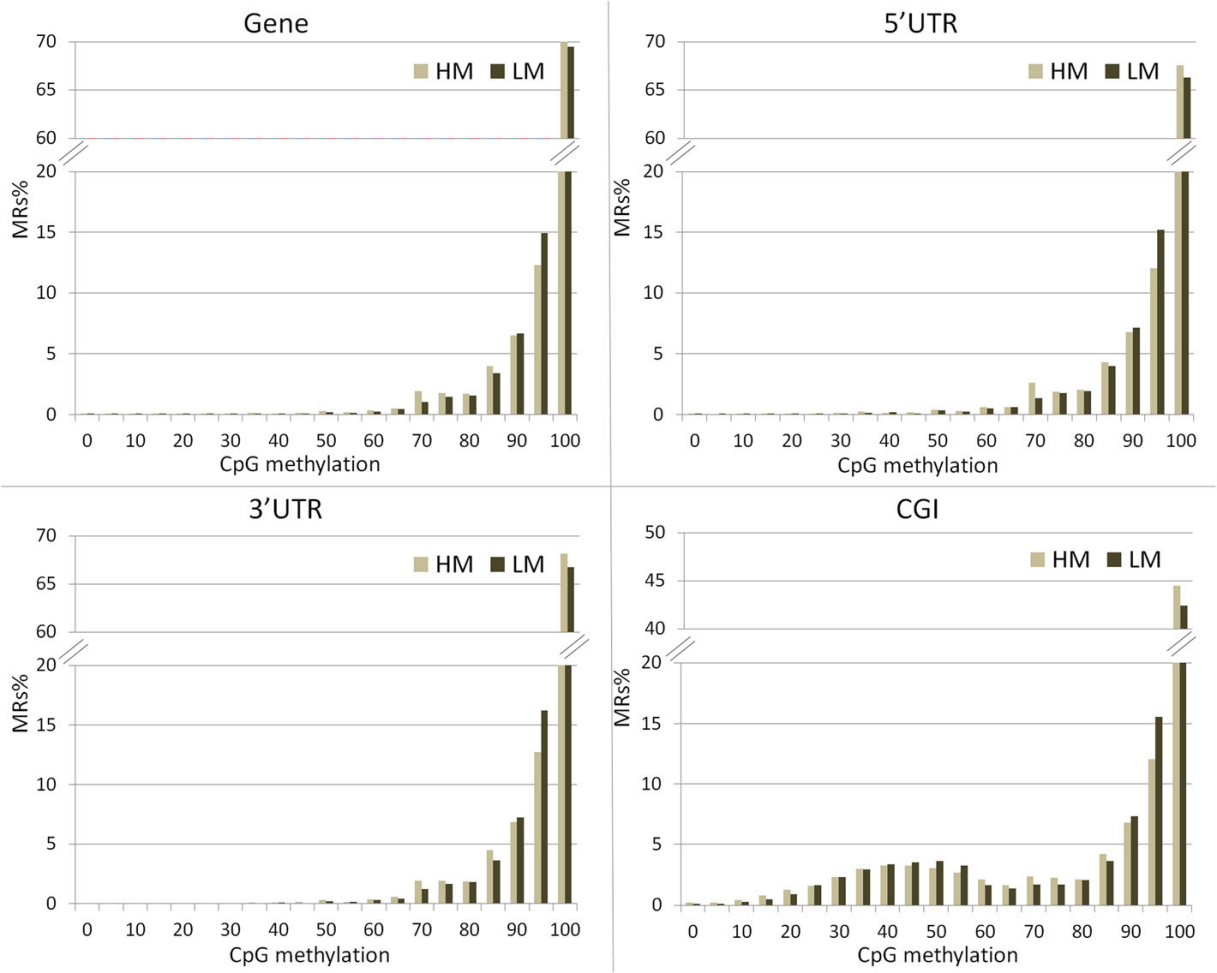
Distribution of methylation sites: gene bodies (GENE), 5’UTR, 3’UTR and CpG island (CGI). Methylated regions (MRs) were stratified based on the average methylation level of CpGs (ranging from 0 to 100%)

Among these, a total of 423,673 MRs mapped in 14,071 out of 23,970 annotated genes. Furthermore, 12,744 MRs mapped upstream (−2Kb) and 19,475 MRs downstream (+2Kb) of gene regions. A total of 9397 MRs were located within the 23,431 annotated CpG islands (CGIs) (Additional files 4, 5, 6 and 7). Gene bodies, 5′ and 3′ UTRs were prevalently hyper-methylated in both sperm populations. Intriguingly, probes overlapping CGIs showed a peculiar distribution, with a relevant proportion of cytosines having an intermediate level of methylation (between 30 and 60%) (Fig. 1).

### Differentially methylated regions between HM and LM sperm populations

**Fig. 2:**
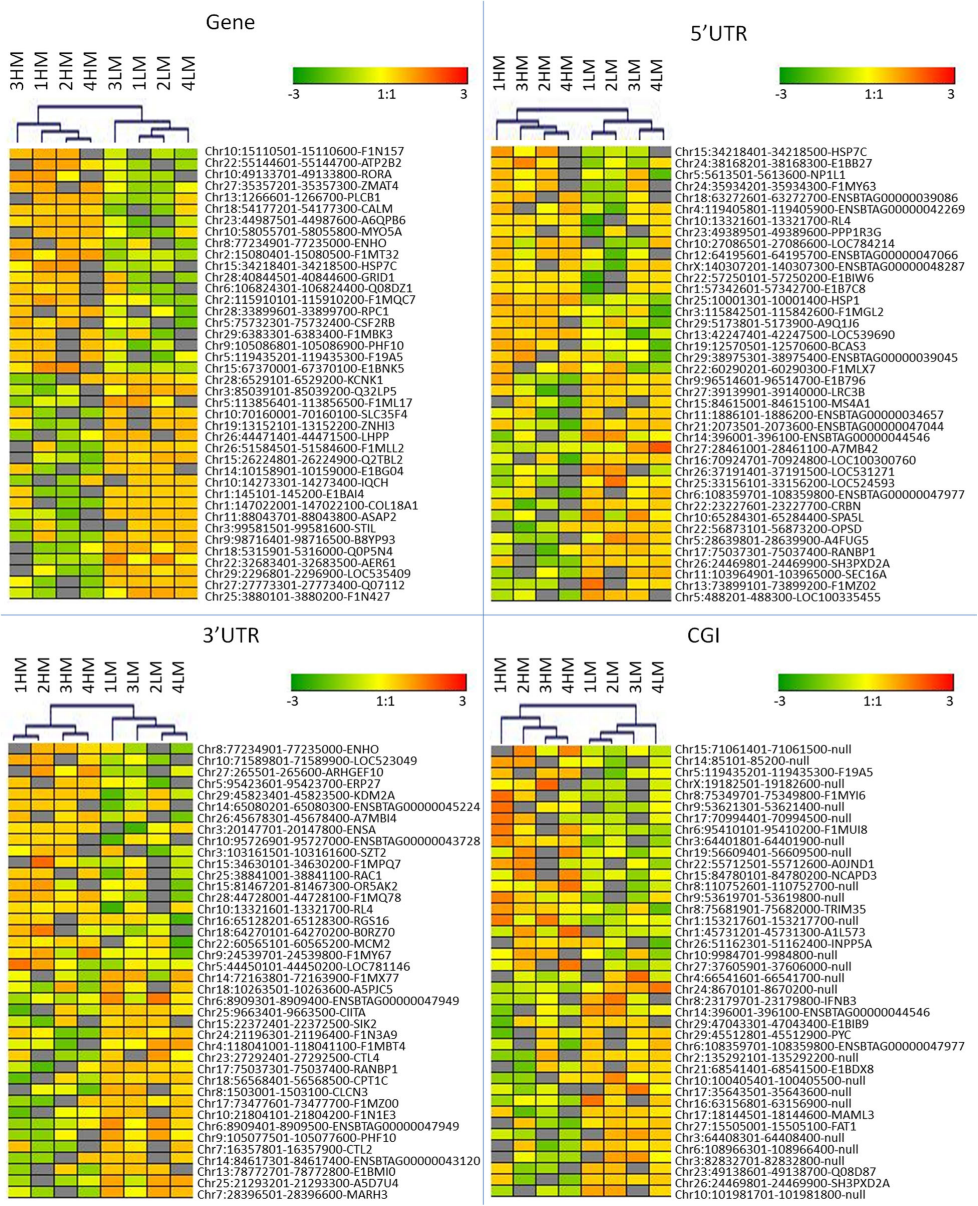
Hierarchical clustering for (DMRs) found in gene bodies, 5’UTR, 3’UTR and CGIs. HM and LM sperm populations were compared and 20 most hyper and 20 most hypo methylated DMRs from each comparison were used for clustering samples

A genome-wide analysis of genes and regulatory elements revealed that a small percentage of CpGs shows a significant variation in the methylation level (differentially methylated regions (DMRs)/MRs percentage) between HM and LM sperm populations in gene bodies (1.45%), 5′ untranslated regions (5’UTRs) (3.12%) and 3’UTRs (2.72%). Considering CGIs, a higher proportion of the methylome (9.77%) was remodelled in HM vs LM sperm populations. Hierarchical analysis of the 20 most hyper and hypo methylated DMRs found in CGIs, in gene bodies, 5’UTR and 3’UTR well discriminated HM from LM samples (Fig. 2). Annotation of 6131 DMRs that overlapped gene bodies resulted in 3278 differentially methylated genes (DMGs) (Additional file 8). In addition, 398, 538 and 918 DMRs located near 5’UTR, 3’UTRs and CGIs, were close (± 2Kb) to 355, 484 and 297 DMGs, respectively (Additional files 9, 10 and 11). Gene ontology (GO) analysis was performed on genes found to be differentially methylated in 5’UTR, 3’UTRs and CGIs, and on a selection of 423 genes differentially methylated in gene bodies (468 DMRs with false discovery rate (FDR) < 10exp-10). Variation in CpG methylation in different gene features and CGIs affect GO terms related to DNA replication, repair, and DNA and telomere organization and maintenance. In addition, GO terms related to hindbrain function, epithelia and endothelia migration metabolic processes were also observed to differ between HM and LM sperm populations. Unexpectedly, a large part of significant gene ontology terms are related to 3’UTRs, whereas only few terms are affected by CpG variation in 5’UTR (Table 1).

**Table 1:** GO terms identified for the differentially methylated genes (DMGs) found to differ between high motile (HM) and low motile (LM) sperm populations in gene bodies (GENE), 5′ untranslated regions (5’UTRs), 3′ untranslated regions (3’UTRs) and CpG islands (CGIs)

GO-ID	GO-Term	Associated Genes Found	*P*-Value*
GENE			
GO:0000723	Telomere maintenance	[ERCC4, LRIG1, PRKDC, TEP1, WRN]	1.88E-02
GO:0032200	Telomere organization	[ERCC4, LRIG1, PRKDC, TEP1, WRN]	1.92E-02
GO:0019722	Calcium, mediated multicellular organism signaling	[ASPH, HDAC4, ITPR1, KSR2, P2RX3, PLCE1]	2.13E-02
GO:0033555	Response to stress	[CACNA1B, GRIN2B, P2RX3]	2.46E-02
GO:0043954	cellular component maintenance	[ABL2, MTMR2, MTSS1]	2.81E-02
GO:0021575	Hindbrain morphogenesis	[ABL2, ATP2B2, DLC1, LDB2]	3.48E-02
GO:0031623	Receptor internalization	[CAV3, MTMR2, PICALM]	4.76E-02
5’UTR			
GO:0010634	Positive regulation of epithelial cell migration	[BCAR1, BCAS3, ENPP2, WNT7A]	3.70E-03
GO:0010595	Positive regulation of endothelial cell migration	[BCAR1, BCAS3, WNT7A]	5.16E-03
3’UTR			
GO:0035162	Embryonic haemopoiesis	[GATA3, KAT6A, KMT2A, PBX1, STK4]	1.31E-04
GO:0006516	Glycoprotein catabolic process	[FBXO6, GPC1, NEU4]	4.45E-03
GO:0046470	Phosphatidylcholine metabolic process	[LIPC, LPCAT3, PLA2G2E, SLC44A2, SLC44A4]	1.11E-02
GO:0051569	Regulation of histone H3-K4 methylation	[AUTS2, GATA3, KMT2A]	2.37E-02
GO:1901616	Organic hydroxy compound catabolic process	[IMPA1, LIPC, NUDT3]	2.83E-02
GO:0042439	Ethanolamine, containing compound metabolic process	[LIPC, LPCAT3, PLA2G2E, SLC44A2, SLC44A4]	3.05E-02
GO:0006303	Double, strand break repair via nonhomologous end joining	[KDM2A, PRPF19, WHSC1]	3.85E-02
GO:0071353	Cellular response tointerleukin-4	[GATA3, MCM2, MCM7]	4.24E-02
GO:0032508	DNA duplex unwinding	[FBXO18, MCM2, MCM7, MRPL36]	4.29E-02
GO:0032392	DNA geometric change	[FBXO18, MCM2, MCM7, MRPL36]	4.31E-02
GO:0000726	non-recombinational repair	[KDM2A, PRPF19, WHSC1]	4.97E-02
CGI			
GO:0035637	Multicellular organismal signaling	[CACNA1C, DMRT3, DPP6, KCNQ1, NFASC, P2RX3]	8.53E-04
GO:0032288	Myelin assembly	[GPC1, NFASC, TENM4]	1.30E-03
GO:0006942	regulation of striated muscle contraction	[CACNA1C, KCNQ1, PDE5A, TNNT3]	3.22E-03
GO:0019226	transmission of nerve impulse	[DMRT3, DPP6, NFASC, P2RX3]	3.62E-03
GO:0032200	Telomere organization	[ERCC4, LRIG1, TERT, WRN]	3.80E-03
GO:0000723	Telomere maintenance	[ERCC4, LRIG1, TERT, WRN]	5.45E-03

Indicated are gene ontology IDs (GO-ID), gene ontology terms (GO-term), associated genes found and corrected *p*-values as determined by ClueGO (http://apps.cytoscape.org/apps/cluego)

* Term *P*-Value Corrected with Bonferroni step down

### Methylation distribution in CpG islands

**Fig. 3:**
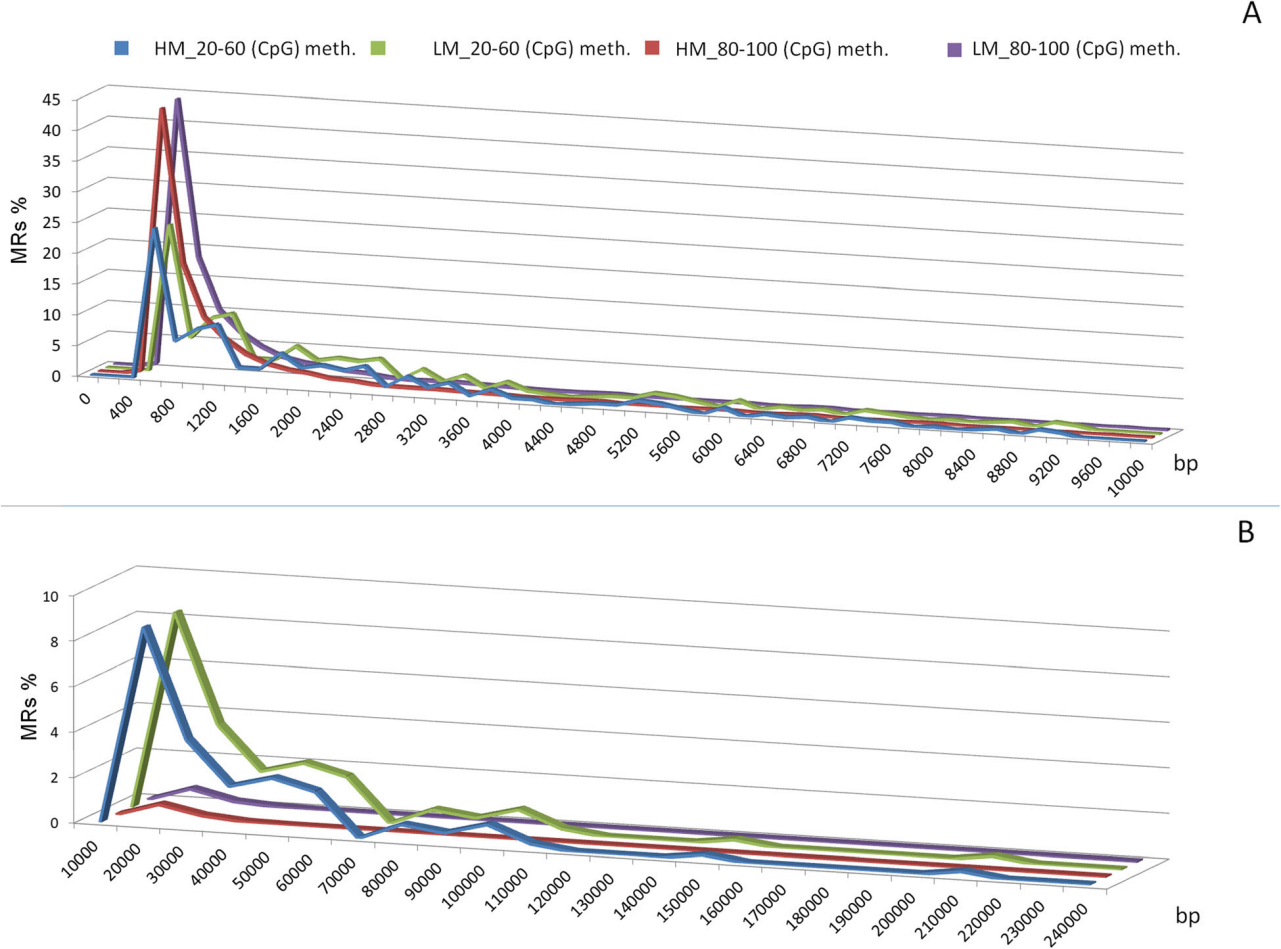
Distribution of CGIs length for Methylated regions (MRs) grouped based on CpG methylation level for HM and LM sperm populations. Methylated regions (MRs) were stratified based on the average methylation level of CpGs (ranging from 0 to 100%). **a** CGIs size between 0 and 10 Kbp, **b** CGIs size between 10 and 240 Kbp

To further explore bovine sperm CpG methylation in CGIs, the global level of cytosine methylation was calculated in each CGI. Out of 23,431 CGIs annotated in the bovine genome, 3869 were detected (in at least 3 out of 4 samples for HM and LM) in our dataset. Based on CpG methylation level in CGIs (Fig. 1), profiles were grouped in two classes (20–60% and 80–100%), and distribution of CGIs length was calculated in each class in HM and LM sperm populations (Fig. 3). Differences in length distribution can be observed between the two classes, both in short (4–10 Kb, Fig. 3a) and long (10–240 Kb, Fig. 3b) CGIs. Notably, 20–60% methylated CGIs exhibit peaks in length distribution that are not observable in highly methylated ones. This behaviour supports the hypothesis that genomic elements (i.e. repetitive elements motifs) present in single or multiple copies could be the target for methylation at low/intermediate levels. In particular, the 1400-bp peak observable in Fig. 3a is compatible with the expected length of Bovidae alpha satellites centromere repeats [29, 30].

### Methylation distribution in repetitive DNA elements

Analysis of CGIs size distribution in low/intermediate methylated regions suggests that the atypical methylation profile observed is likely associated to repetitive DNA elements.

To further test this hypothesis, low/intermediate and highly methylated sequences were used as a query to the Database of repetitive rDNA elements Repbase. Database interrogation returned the bovine satellite BTSAT4 for about 75% of intermediate methylated sequences, whereas the percentage of BTSAT4 in hypermethylated regions was close to zero. Other repetitive elements such as BTSAT3 and OSSAT2 satellites and BTLTR1 and ERV2–1-LTR transposable elements were also methylated prevalently at intermediate level (20–60%) (Table 2). Out of 2434 BTSAT4 elements annotated in the bovine genome 720 were detected (in at least 3 out of 4 samples for HM and LM) in our dataset.

**Table 2:** Frequency of occurrence for Repetitive Elements (REs) overlapping CGIs with different methylation levels (20–60% methylation and 80–100% methylation) in high motile (HM) and low motile (LM) sperm populations. Frequency of occurrence for REs is also reported for *Bos taurus* genome

HM				LM				GENOME	
CGIs methyl. 20–60%		CGIs methyl. 80–100%		CGIs methyl. 20–60%		CGIs methyl. 80–100%		Methyl. ref. genome	
RE type	%	RE type	%	RE type	%	RE type	%	RE type	%
BTSAT4	75.3	GC-rich	24	BTSAT4	75.9	GC-rich	23.5	Bov-tA2	8.6
SSU-rRNA	4.5	Bov-tA2	3.8	SSU-rRNA	4.6	Bov-tA2	4.1	ART2A	8.3
BTSAT2	3.8	MIRb	3.4	BTSAT2	3.9	MIRb	3.3	BovB	6.7
GC-rich	3	ART2A	2.6	GC-rich	3.3	C-rich	2.7	BOV-A2	5.1
OSSAT2	1.7	(TG)n	2.6	OSSAT2	1.7	ART2A	2.7	AT-rich	4.9
BTLTR1	1.2	C-rich	2.6	BTLTR1	1.2	(TG)n	2.6	Bov-tA1	3.7
ERV2–1-LTR	0.9	(CA)n	2.2	ERV2–1-LTR	0.9	(CA)n	2.2	MIRb	3.3
5S-rRNA	0.9	Bov-tA1	1.9	BTSAT3	0.8	Bov-tA1	2	MIR	2.5
BTSAT3	0.7	MIR	1.9	5S-rRNA	0.7	CHR-2A	1.8	L2a	2.2
LSU-rRNA	0.6	CHR-2A	1.8	LSU-rRNA	0.5	BovB	1.8	L1–2	2.1
G-rich	0.4	G-rich	1.7	BovB	0.4	MIR	1.7	L1	1.8
(CA)n	0.4	BovB	1.6	G-rich	0.4	BOV-A2	1.7	L2c	1.6
(CGGGG)n	0.4	BOV-A2	1.6	(CGGGG)n	0.4	G-rich	1.6	Bov-tA3	1.6
Bov-tA2	0.3	L2b	1.5	(CA)n	0.4	L2b	1.5	BTLTR1	1.5
BovB	0.3	L1–2	1.5	C-rich	0.3	L1–2	1.5	L2b	1.3
(TG)n	0.3	MIR3	1.4	(TG)n	0.2	CHR-2B	1.4	MIRc	1.2
(CGTG)n	0.3	CHR-2B	1.3	(CCCCAG)n	0.2	MIR3	1.4	MIR3	1.2
Bov-tA3	0.3	MLT1B	1.1	(CGG)n	0.2	MIRc	1.1	L1	1.2
C-rich	0.3	MIRc	1.1	L1–3	0.2	MLT1B	1.1	L1–3	1.1

**Fig. 4:**
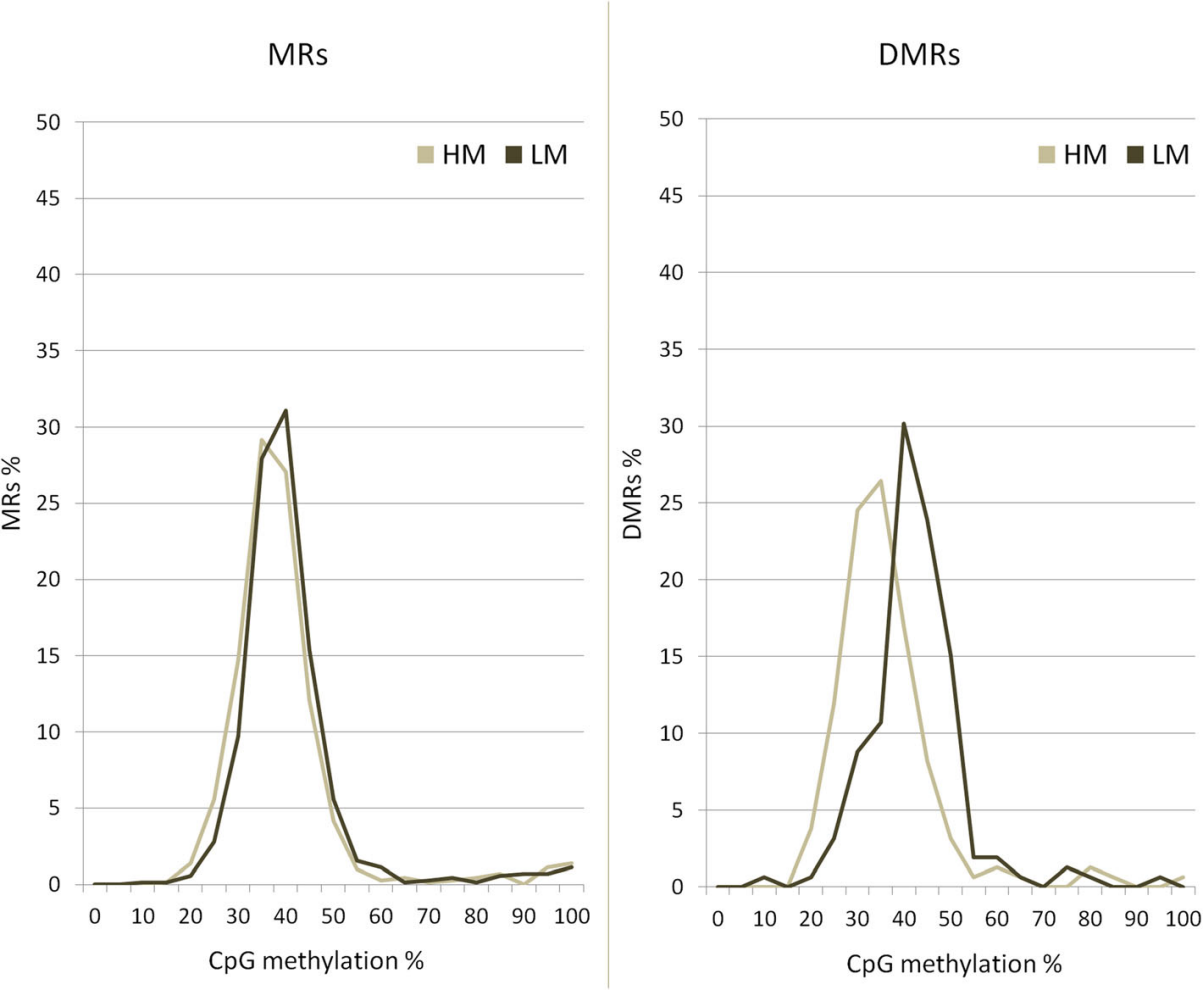
Distribution of methylation sites in BTSA4. Methylated regions (MRs), left panel, and differentially methylated region (DMRs), right panel, were stratified based on the average methylation level of CpGs (ranging from 0 to 100%) for HM and LM sperm populations

Analysis of CpG methylation outlined an overall low level of BTSAT4 methylation in the HM sperm population. Considering 159 DMRs in the BTSAT4 regions, 122 were more methylated in LM sperm populations (Additional file 12) (Fig. 4).

To further support the hypothesis of a targeted methylation pattern of repetitive DNA elements, the genomic distribution of CGIs and BTSAT4 elements was observed. The overall CGIs covered by MBD sequencing represent about 1% of the total cow genome, while sequenced BTSAT4 regions represent 0.2%. The intersection of genomic absolute positions of CGIs and BTSAT4 repeats shows that 70,6% of the BTSAT4 base pairs fall within CGIs, 80% of the BTSAT4 repeats being completely or partially located within CGIs (Additional file 13).

## Discussion

In this work, the pattern of methylation in high and low motile bull sperm populations was determined using an enrichment step of methyl-CpG sequences combined with bisulfite sequencing.

The comparison of different genomic features in HM and LM sperm populations revealed several differentially methylated regions flanking genes with a role in chromatin organization and maintenance. Undoubtedly, a correlation between sperm telomere length and abnormal sperm parameters exists [31]. Interestingly, HM and LM sperm populations showed variation in methylation of telomerase reverse transcriptase (TERT) and telomerase-associated protein 1 (TEP1). Genetic variants in both genes were previously reported to be associated with susceptibility to male infertility [32].

Differential methylation in 3’UTRs was also found in genes (histone lysine methyltransferases 2A (KMT2A), histone lysine demethylases 2A (KDM2A) and nuclear receptor-binding SET Domain 2 (NSD2)/ multiple myeloma SET domain (MMSET)/ Wolf-Hirschhorn syndrome candidate-1(WHSC1)) influencing chromatin structure by epigenetic mechanisms, such as the regulation of histone H3-K4 methylation. Previous studies reported a strict association between sperm DNA methylation levels and both sperm chromatin condensation and DNA integrity, suggesting that the formation of a compact chromatin and proper DNA methylation are closely related events during spermatogenesis [21].

The NSD family of histone methyltransferase (HMT) comprises three members (NSD1, NSD2/ MMSET/ WHSC1, and NSD3/WHSC1L) that recognise lysine residue of histones H3 and H4 and mediate their methylation [33]. KMT2A (also known as mixed-lineage leukemia 1 (MLL1)) catalyzes the methylation of H3K4 [34, 35]. KDM2A, a Jumonji-C (JmjC)-domain containing histone demethylase (HDM), is a heterochromatin-associated protein that is required to maintain the heterochromatic state, it represses transcription of small non-coding RNAs that are encoded by clusters of satellite repeats at the centromere [36].

Histone Lysine methylation is tightly regulated by distinct families of conserved enzymes, KMTs and KDMs, which add and remove methyl groups at histone lysines [37]. They play a role in orchestrating methylation of H3K9 and H3K27 in sperm. The methylation increases during meiosis, but the removal of H3K9me at the end of meiosis is essential for the onset of spermiogenesis [38]. In mice, the reduction of MLL2 activity results in a dramatic decrease of the number of spermatocytes by an apoptotic process and prevents spermatogenic differentiation [39]. Lysine-specific histone demethylase 1 (LSD1)/KDM1 is required for spermatogonial differentiation, as well as germ cell survival, in the developing testis [40]. An evolutionarily conserved pathway between histone H3-K9 methylation and DNA methylation exists in mammals, that is likely to be important to reinforce heterochromatic subdomains stability and to protect genome integrity. Suppressor of variegation 3–9 homolog (Suv39h) HMTases (also called KMT1A/B) are required to direct H3-K9 trimethylation and DNA (cytosine-5-)-methyltransferase 3 beta (Dnmt3b)-dependent DNA methylation to major satellite repeats at pericentric region [41].

A concurring variation in methylation of satellite repeats at pericentric region was observed in our dataset. A group of CGIs methylated at intermediate level (20–60%), located within genomic satellite repeats and in particular BTSAT4 Bovine satellite I [29] was observed to be less methylated in HM sperm populations. In the bovine genome BTSAT4 is likely to be the counterpart of human alpha satellites, because both present in high-copy tandem repeat at centromeric position. Comparative analysis in hundreds of species shows a high variability in size for alpha satellites centromere repeats, e.g. approximately 171-bp in human and 1400-bp in Bovidae [30], which is in agreement with the size of repetitive element that we found to be methylated at intermediate level in bovine sperm. The location of bovine satellite I in all pericentric regions of *Bos taurus* autosomes was also confirmed by fluorescence in situ hybridization [42].

In accordance with our study, a low level of methylation of satellite DNA within pericentric regions was previously observed in primate sperm profiling [13]. Bovine alpha satellite I was observed to have low/intermediate methylation levels in sperm. Embryos obtained by somatic cell nuclear transfer (SCNT) presented a hyper-methylation in the bovine alpha satellite I, expected to cause higher chromatin condensation compared to embryos generated by in vitro sperm fertilization (IVF). This may in turn contribute, either immediately or later in development, to the inefficiency of producing live offspring by SCNT [43, 44]. Low methylation levels have been also correlated with the ability to bind cohesin complexes that regulate the separation of chromatids at mitosis [45], suggesting a model in which selective hypomethylation of centromeric satellites might be critical for accurate chromosome segregation during meiosis. Recently, methylation at satellite repeats throughout the genome has been observed to be increased in obese rat offspring [46]. Although obesity in human is associated with infertility by numerous studies [47], a direct link between satellite repeats methylation and sperm infertility is not yet described.

## Conclusion

Methylation profiling in bovine semen revealed differential methylation of the BTSAT4 repetitive element in pericentric regions between HM and LM sperm populations. In addition, many DMRs were enriched in genes often functionally related to sperm DNA organization and maintenance. Together, alteration of methylation in satellite regions within the pericentric regions and in genes associated to lysine histone methylation, highlight that the complex mechanism that regulates DNA condensation during chromosomal packaging in sperm may affect sperm motility.

## Methods

### Isolation of spermatozoa and evaluation of sperm motility

Frozen semen straws from four mature progeny tested Holstein bulls with satisfactory semen quality were purchased from an Artificial Insemination AI center (INSEME S.P.A., Modena, Italy).

High Motile (HM) and Low Motile (LM) sperm populations were isolated through Percoll gradient as previously described [48]. Total motility and sperm kinetics parameters were assessed by CASA (Computer-Assisted Semen Analysis) system (ISAS® v1). Five μl of semen pellet obtained after Percoll density gradient centrifugation were diluted in 5 μl Tyrode’s albumine lactate pyruvate (TALP) sperm medium [49] pre-warmed at 37 °C. Ten μl of diluted semen was placed on a pre-warmed (37 °C) Makler chamber. During the analysis, the microscope heating stage was maintained at 37 °C. Using a 10× objective in phase contrast, the image was relayed, digitized and analyzed by the ISAS® software with user-defined settings as follows: frames acquired, 25; frame rate, 20 Hz; minimum particles area 20 μm^2^; maximum particles areas 70 μm^2^. Spermatozoa speed was assigned to 3 broad categories: rapid (50 μm/s), medium (25 μm/s) and slow (10 μm/s). CASA kinetics parameters were: total motility (MOT TOT, %), progressive motility (PRG, %), curvilinear velocity (VCL, μm/s), straight-line velocity (VSL, μm/s), average path velocity (VAP, μm/s), linearity coefficient (LIN, %, = VSL/VCL × 100), amplitude of lateral head displacement (ALH, μm), straightness coefficient (STR, % = VSL/VAP × 100), wobble coefficient (WOB, % = VAP/VCL × 100) and beat cross frequency (BCF, Hz).

#### DNA extraction, library preparation and sequencing

Four HM and four LM sperm samples obtained in previous step were used for DNA extraction. DNA was isolated by NucleoSpin® Tissue (Macherey-Nagel) following manufacturer instruction. One μg of genomic DNA was sonicated to produce DNA fragments of about 350 bp lengths. Methyl-binding domain (MBD) enrichment was performed using the MethylMiner™ Methylated DNA Enrichment Kit (Thermo Fisher Scientific), following manufacture instruction. Libraries were generated using the TruSeq® DNA PCR-Free Library Preparation Kit (Illumina) including a step of bisulfite treatment. After adapters ligation, samples were converted with EpiTect Bisulfite Kits (Qiagen) and finally PCR amplified with KAPA HiFi Uracil+ (Kapa Biosystems) to obtain methyl enriched bisulfite libraries. The eight libraries were used for cluster generation and subsequently sequenced on a single lane of Illumina Hiseq2000.

#### Statistical analysis and bionformatics

Data obtained from CASA were analyzed using the SAS™ package v 9.4 (SAS Institute Inc.). The General Linear Model procedure (PROC GLM) was used to evaluate the efficiency of the sperm separation comparing semen quality parameters at thawing and in the HM population. The model included the fixed effect of the sperm population, and bull as random. Results are given as adjusted least squares means ± standard error means (LSM ± SEM).

Preliminary quality control of raw reads was carried out with FastQC (http://www.bioinformatics.babraham.ac.uk/projects/fastqc/). Illumina raw sequences were then filtered with Trimmomatic [50] to remove adapters and low quality bases at the ends of sequence, using a sliding window approach. Data are available in the Sequence Reads Archive (SRA), (Accession Number SRP119411). Bismark software v.0.17.0 (https://www.bioinformatics.babraham.ac.uk/projects/bismark/) was used to align each read to a bisulfite-converted *Bos taurus* genome UMD311 with option -N 1, and methylation calls were extracted using the Bismark methylation_extractor function. Seqmonk software (version 0.34.1) was used for visualization and analysis of the Bismark output (http://www.bioinformatics.babraham.ac.uk/projects/seqmonk/). Only position with at least 5 cytosine were recorded in all samples, others were discarded from the data set. Cytosines count for each position (*n* = 1,742,816, count>5X, present at least in 3 out of 4 in HM and LM samples) was determined and the Edge-R package was used to evaluate if over or under-representation occurs in our dataset between HM and LM groups.

Methylated regions (MRs) were detected genome wide by dividing the genome in 100 bp tiles and analyzing average methylation in a sliding window of 100 bp. MRs were considered if present at least in 3 out of 4 samples in both HM and LM sperm populations. Methylation was calculated independently for different features: 5′ UTR (−2Kb), 3′ UTR (+2Kb), gene bodies and CpG islands (CGIs). MRs were also determined per CGI length classes and overlapping BTSAT4 REs. Reads count in BTSAT4 REs was determined and the Edge-R package was used to evaluate if over or under-representation occurs in our dataset between HM and LM groups. Differentially methylated regions (DMRs) between HM and LM populations were calculated using the logistic regression filter in R to assess differential methylation (FDR < 0.05, absolute cut-off of 5%). Hierarchical clustering was produced for DMRs present in CGIs, gene bodies, 5′ UTRs and 3′ UTRs. The level of methylation was normalized across samples and methylation percentage from a selection of DMRs showing the highest differences in methylation was used for clustering using the Genesis software [51].

Genes included in DMRs at CGIs and different genomic features were submitted to GO analysis. GO classification of the DMRs was performed according to canonical GO categories, using the Cytoscape plug-in ClueGO which integrates GO [52] and enhances biological interpretation of large lists of genes. Evaluation of REs in CGIs was performed by intersecting genomic positions of both features by Bedtools intersect (http://bedtools.readthedocs.io), thus frequencies for each RE category were calculated for low/intermediate methylation CGIs (20–60% methylation) and high methylation CGIs (80–100% methylation), in both HM and LM sperm populations and in *Bos taurus* genome.

## Supplementary information

**Additional file 1. **Kinetics parameters evaluated on semen at thawing and in High Motile population: MOT TOT total motility, PRG cells progressive motility, VSL straight-line velocity, VCL curvilinear velocity, VAP average path velocity, LIN linear coefficient, STR straightness coefficient, WOB wobble coefficient, ALH amplitude of lateral head displacement, BCF beat cross-frequency. Results are given as adjusted least squares means ± standard error means (LSM ± SEM). a,b values within a row with different superscripts differ significantly at *P* < 0.05.

**Additional file 2.** Edge-R smear plot representing the Average Log Count Per Millions (CPM) and the abundance differences (logFC = log Fold Change) for the cytosine counts between HM and LM groups. Not significant differences (False Discovery Rate FDR < 0.05) between the two groups were observed (in red).

**Additional file 3.** Bismark sequencing statistics for the four replicates (1–4) of high motile (HM) and low motile (LM) sperm population.

**Additional file 4.** Methylated Regions (MRs) found at least in three samples in both high motile (HM) and low motile (LM) sperm populations overlapping gene bodies. Columns report: Probe name, position (Chromosome, Start, End), Feature, protein ID, Description, MRs percentage for each sample (1HM, 2HM, 3HM, 4HM, 1LM, 2LM, 3LM, 4LM).

**Additional file 5.** Methylated Regions (MRs) found at least in three samples in both high motile (HM) and low motile (LM) sperm populations upstream of genes (5’UTR). In each column are reported: Probe name, position (Chromosome, Start, End), Feature, protein ID, Description, MRs percentage for each sample (1HM, 2HM, 3HM, 4HM, 1LM, 2LM, 3LM, 4LM).

**Additional file 6.** Methylated Regions (MRs) found at least in three samples in both high motile (HM) and low motile (LM) sperm populations downstream of genes (3’UTR). In each column are reported: Probe name, position (Chromosome, Start, End), Feature, protein ID, Description, MRs percentage for each sample (1HM, 2HM, 3HM, 4HM, 1LM, 2LM, 3LM, 4LM).

**Additional file 7.** Methylated Regions (MRs) found at least in three samples in both high motile (HM) and low motile (LM) sperm populations overlapping CpG islands (CGIs). In each column are reported: Probe name, position (Chromosome, Start, End), Feature, protein ID, Description, MRs percentage for each sample (1HM, 2HM, 3HM, 4HM, 1LM, 2LM, 3LM, 4LM).

**Additional file 8.** Differentially Methylated Regions (DMRs) found at least in three samples in both high motile (HM) and low motile (LM) sperm populations overlapping gene bodies. In each column are reported: Probe name, position (Chromosome, Start, End), False Discovery Rate (FDR), Feature, protein ID, Description, MRs percentage for each sample (1HM, 2HM, 3HM, 4HM, 1LM, 2LM, 3LM, 4LM).

**Additional file 9.** Differentially Methylated Regions (DMRs) found at least in three samples in both high motile (HM) and low motile (LM) sperm populations upstream of genes (5’UTR). In each column are reported: Probe name, position (Chromosome, Start, End), False Discovery Rate (FDR), Feature, protein ID, Description, MRs percentage for each sample (1HM, 2HM, 3HM, 4HM, 1LM, 2LM, 3LM, 4LM).

**Additional file 10.** Differentially Methylated Regions (DMRs) found at least in three samples in both high motile (HM) and low motile (LM) sperm populations downstream of genes (3’UTR). In each column are reported: Probe name, position (Chromosome, Start, End), False Discovery Rate (FDR), Feature, protein ID, Description, MRs percentage for each sample (1HM, 2HM, 3HM, 4HM, 1LM, 2LM, 3LM, 4LM).

**Additional file 11.** Differentially Methylated Regions (DMRs) found at least in three samples in both high motile (HM) and low motile (LM) sperm populations overlapping CpG islands (CGIs). In each column are reported: Probe name, position (Chromosome, Start, End), False Discovery Rate (FDR), Feature, protein ID, Description, MRs percentage for each sample (1HM, 2HM, 3HM, 4HM, 1LM, 2LM, 3LM, 4LM).

**Additional file 12.** Differentially Methylated Regions (DMRs) found at least in three samples in both high motile (HM) and low motile (LM) sperm populations overlapping BTSAT4 satellite. In each column are reported: Probe name, position (Chromosome, Start, End), False Discovery Rate (FDR), Feature, protein ID, Description, MRs percentage for each sample (1HM, 2HM, 3HM, 4HM, 1LM, 2LM, 3LM, 4LM, average HM, average LM and differences between averages Δ HM-LM).

**Additional file 13. **Statistics for the distribution of CpG islands (CGIs) and repetitive element BTSAT4 satellite along the *Bos taurus* genome. The intersections of CGIs and BTSAT4 regions in function of length (bps), number (nr) and percentage (%) are reported.

## Data Availability

All sequence data are deposited at the NCBI Sequence Read Archive (SRA) (https://www.ncbi.nlm.nih.gov/sra) (Accession Number SRP119411).

## Abbreviations

5-mC: 5 methyl cytosine
ALH: Amplitude of lateral head displacement
BCF: Beat cross frequency
CGIs: CpG islan ds
CpGs: Cytosine-guanine dinucleotides
DMG: Differentially methyalted gene
DMR: Differentially methyalted region
FDR: False discovery rate
GO: Gene ontology
HDMase: Histone demethylase
HM: High motile
HMTases: Histone methylases
IVF: In vitro fertilization
KDMs: Histone lysine demethylases
KMTs: Histone lysine methyltransferases
LIN: Linearity coefficient
LINE: Long interspersed elements
LM: Low motile
MBD: Methyl-binding domain
MOT TOT: Total motility
MR: Methylated region
PRG: Progressive motility
RE: Repetitive element
RRBS: Reduced Representation Bisulfite Sequencing
SCNT: Somatic cell nuclear transfer
STR: Straightness coefficient
VAP: Average path velocity
VCL: Curvilinear velocity
VSL: Straight-line velocity
WOB: Wobble coefficient
