# Supplementary material for: Epigenetic analysis of high and low motile sperm populations reveals methylation variation in satellite regions within the pericentromeric position and in genes functionally related to sperm DNA organization and maintenance in Bos taurus
Source: BMC Genomics. 2019 Dec 6;20:940. doi: 10.1186/s12864-019-6317-6 (PMC6898967; doi:10.1186/s12864-019-6317-6)
Supplement: Supplementary file 1 — Additional file 1. Kinetics parameters evaluated on semen at thawing and in High Motile population: MOT TOT total motility, PRG cells progressive motility, VSL straight-line velocity, VCL curvilinear velocity, VAP average path velocity, LIN linear coefficient, STR straightness coefficient, WOB wobble coefficient, ALH amplitude of lateral head displacement, BCF beat cross-frequency. Results are given as adjusted least squares means ± standard error means (LSM ± SEM). a,b values within a row with different superscripts differ significantly at P < 0.05. [file 12864_2019_6317_MOESM1_ESM.docx]

|  | **Semen at thawing** | **High Motile population** |
| --- | --- | --- |
| **MOT TOT (%)** | 76.24±11.10 | 74.19±7.85 |
| **PRG (%)** | 47.58±6.31 | 45.64±4.47 |
| **VSL (μm/s)** | 46.08±4.11a | 61.24±2.91b |
| **VCL (μm/s)** | 76.35±6.02a | 110.37±4.25b |
| **VAP (μm/s)** | 55.38±4.27a | 74.02±3.01b |
| **LIN (%)** | 60.64±3.07 | 55.05±2.18 |
| **STR (%)** | 83.11±3.08 | 82.63±2.18 |
| **WOB (%)** | 72.74±1.48a | 66.68±1.05b |
| **ALH (μm)** | 2.53±0.14a | 3.72±0.10b |
| **BCF (Hz)** | 8.67±0.45 | 8.69±0.32 |

**Additional file 1.** Kinetics parameters evaluated on semen at thawing and in High Motile population: MOT TOT total motility, PRG cells progressive motility, VSL straight-line velocity, VCL curvilinear velocity, VAP average path velocity, LIN linear coefficient, STR straightness coefficient, WOB wobble coefficient, ALH amplitude of lateral head displacement, BCF beat cross-frequency. a,b values within a row with different superscripts differ significantly at P <0.05.
