# Supplementary material for: Epigenetic analysis of high and low motile sperm populations reveals methylation variation in satellite regions within the pericentromeric position and in genes functionally related to sperm DNA organization and maintenance in Bos taurus
Source: BMC Genomics. 2019 Dec 6;20:940. doi: 10.1186/s12864-019-6317-6 (PMC6898967; doi:10.1186/s12864-019-6317-6)
Supplement: Supplementary file 2 — Additional file 2. Edge-R smear plot representing the Average Log Count Per Millions (CPM) and the abundance differences (logFC = log Fold Change) for the cytosine counts between HM and LM groups. Not significant differences (False Discovery Rate FDR < 0.05) between the two groups were observed (in red). [file 12864_2019_6317_MOESM2_ESM.docx]

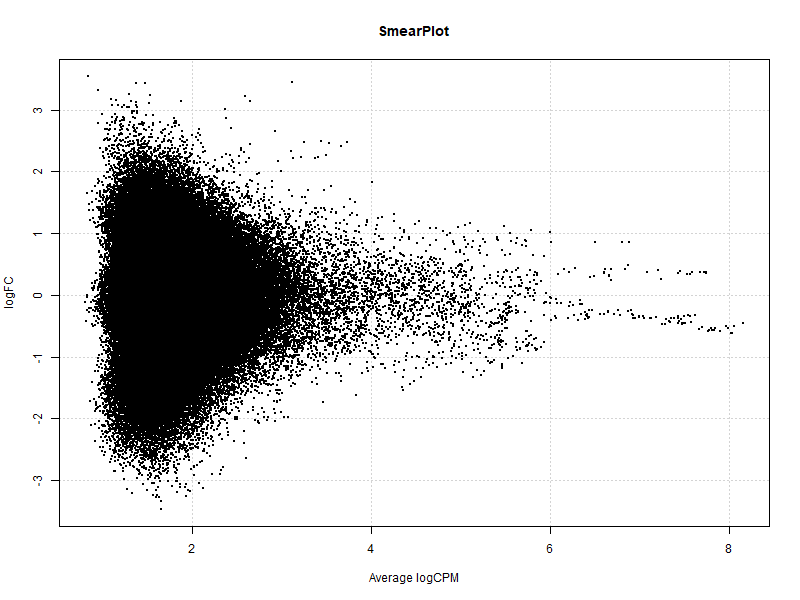


**Additional file 2.** Edge-R smear plot representing the Average Log Count Per Millions (CPM) and e the abundance differences (logFC= log Fold Count) for the cytosine counts between HM and LM groups. Not significative differences (False Discovery Rate FDR<0.05) between the two groups were observed (in red).
