# Supplementary material for: Epigenetic analysis of high and low motile sperm populations reveals methylation variation in satellite regions within the pericentromeric position and in genes functionally related to sperm DNA organization and maintenance in Bos taurus
Source: BMC Genomics. 2019 Dec 6;20:940. doi: 10.1186/s12864-019-6317-6 (PMC6898967; doi:10.1186/s12864-019-6317-6)
Supplement: Supplementary file 3 — Additional file 3. Bismark sequencing statistics for the four replicates (1–4) of high motile (HM) and low motile (LM) sperm population. [file 12864_2019_6317_MOESM3_ESM.docx]

|  | **HM1** | **HM2** | **HM3** | **HM4** | **LM1** | **LM2** | **LM3** | **LM4** |
| --- | --- | --- | --- | --- | --- | --- | --- | --- |
| Sequence pairs analysed in total: | 2.73E+07 | 2.25E+07 | 3.55E+07 | 1.32E+07 | 3.04E+07 | 2.78E+07 | 3.06E+07 | 3.75E+07 |
| Number of paired-end alignments with a unique best hit: | 2.34E+07 | 1.95E+07 | 3.09E+07 | 1.10E+07 | 2.71E+07 | 2.42E+07 | 2.66E+07 | 3.39E+07 |
| Mapping efficiency: | 85.60% | 86.60% | 87.10% | 83.10% | 89.10% | 87.10% | 86.80% | 90.60% |
| Sequence pairs with no alignments under any condition: | 3.31E+06 | 2.26E+06 | 3.90E+06 | 1.88E+06 | 2.68E+06 | 2.84E+06 | 3.44E+06 | 2.28E+06 |
| Sequence pairs did not map uniquely: | 6.31E+05 | 7.56E+05 | 6.81E+05 | 3.47E+05 | 6.27E+05 | 7.60E+05 | 6.06E+05 | 1.26E+06 |
| Total number of C's analysed: | 9.63E+08 | 7.92E+08 | 1.23E+09 | 4.65E+08 | 1.17E+09 | 9.98E+08 | 1.10E+09 | 1.44E+09 |
| Total methylated C's in CpG context: | 1.01E+08 | 7.71E+07 | 1.04E+08 | 5.19E+07 | 1.26E+08 | 9.86E+07 | 1.10E+08 | 1.65E+08 |
| Total methylated C's in CHG context: | 2.16E+06 | 1.25E+06 | 1.97E+06 | 6.72E+05 | 1.26E+06 | 1.03E+06 | 1.54E+06 | 2.80E+06 |
| Total methylated C's in CHH context: | 4.86E+06 | 2.99E+06 | 5.12E+06 | 1.55E+06 | 2.88E+06 | 2.52E+06 | 3.64E+06 | 6.28E+06 |
| Total methylated C's in Unknown context: | 2.67E+02 | 1.79E+02 | 2.19E+02 | 1.34E+02 | 2.91E+02 | 2.18E+02 | 2.65E+02 | 4.46E+02 |
| Total unmethylated C's in CpG context: | 6.76E+06 | 5.56E+06 | 7.12E+06 | 3.75E+06 | 7.90E+06 | 6.49E+06 | 7.40E+06 | 1.08E+07 |
| Total unmethylated C's in CHG context: | 2.50E+08 | 2.03E+08 | 3.09E+08 | 1.21E+08 | 3.05E+08 | 2.56E+08 | 2.83E+08 | 3.76E+08 |
| Total unmethylated C's in CHH context: | 5.98E+08 | 5.02E+08 | 8.00E+08 | 2.86E+08 | 7.22E+08 | 6.33E+08 | 6.92E+08 | 8.79E+08 |
| Total unmethylated C's in Unknown context: | 3.81E+03 | 2.86E+03 | 4.16E+03 | 1.56E+03 | 4.38E+03 | 4.05E+03 | 3.93E+03 | 5.07E+03 |
| C methylated in CpG context: | 93.70% | 93.30% | 93.60% | 93.30% | 94.10% | 93.80% | 93.70% | 93.90% |
| C methylated in CHG context: | 0.90% | 0.60% | 0.60% | 0.60% | 0.40% | 0.40% | 0.50% | 0.70% |
| C methylated in CHH context: | 0.80% | 0.60% | 0.60% | 0.50% | 0.40% | 0.40% | 0.50% | 0.70% |
| C methylated in unknown context (CN or CHN): | 6.50% | 5.90% | 5.00% | 7.90% | 6.20% | 5.10% | 6.30% | 8.10% |

**Additional file 3.** Bismark sequencing statistics for the four replicates (1-4) of high motile (HM) and low motile (LM) sperm populations.
