# Supplementary material for: Epigenetic analysis of high and low motile sperm populations reveals methylation variation in satellite regions within the pericentromeric position and in genes functionally related to sperm DNA organization and maintenance in Bos taurus
Source: BMC Genomics. 2019 Dec 6;20:940. doi: 10.1186/s12864-019-6317-6 (PMC6898967; doi:10.1186/s12864-019-6317-6)
Supplement: Supplementary file 13 — Additional file 13. Statistics for the distribution of CpG islands (CGIs) and repetitive element BTSAT4 satellite along the Bos taurus genome. The intersections of CGIs and BTSAT4 regions in function of length (bps), number (nr) and percentage (%) are reported. [file 12864_2019_6317_MOESM13_ESM.docx]

| Total genome length (chromosomes only) (bps) | 2660922743 |
| --- | --- |
| Total CGIs length (bps) | 26746347 |
| Total BTSAT4 length (bps) | 5321179 |
| BTSAT4 bps intersecting with CGIs (bps) | 3755969 |
| Total nr of BTSAT4 repeats in genome | 2435 |
| Nr of BTSAT4 repeats intersected with CGIs | 1949 |
| Total nr of CGIs in genome | 23431 |
| Nr of CGIs intersecting with BTSAT4 | 567 |
| CGIs bps % of total genome | 1 |
| BTSAT4 bps % of total genome | 0.2 |
| % of CGIs intersecting with BTSAT4 | 2.42 |
| % of BTSAT4 bps intersecting with CGIs | 70.59 |
| % of BTSAT4 repeats intersecting with CGIs | 80 |
|  |  |

**Additional file 13.** Statistics for the distribution of CpG islands (CGIs) and repetitive element BTSAT4 satellite along the *Bos taurus* genome. The intersections of CGIs and BTSAT4 regions in function of length (bps), number (nr) and percentage (%) are reported.
